# Considering the Nature of Multimodal Language from a Crosslinguistic Perspective

**DOI:** 10.5334/joc.165

**Published:** 2021-08-23

**Authors:** Asli Özyürek

**Affiliations:** 1 Donders Institute Brain, Cognition and Behavior, Center for Language Studies, Radboud University and Max Planck Institute for Psycholinguistics, NL

**Keywords:** Embodied cognition, Language production, Word processing

## Abstract

Language in its primary face-to-face context is multimodal (e.g., Holler and Levinson, 2019; Perniss, 2018). Thus, understanding how expressions in the vocal and visual modalities together contribute to our notions of language structure, use, processing, and transmission (i.e., acquisition, evolution, emergence) in different languages and cultures should be a fundamental goal of language sciences. This requires a new framework of language that brings together how arbitrary and non-arbitrary and motivated semiotic resources of language relate to each other. Current commentary evaluates such a proposal by Murgiano et al (2021) from a crosslinguistic perspective taking variation as well as systematicity in multimodal utterances into account.

There is growing consensus in language studies that language in its primary face-to-face context (as is the case both phylogenetically and ontogenetically) is multimodal (e.g., Holler and Levinson, 2019; Perniss, 2018). That is, expressions in the visual modality, such as visible communicative movements (i.e., gestures) universally accompanying spoken languages and sign languages of Deaf Communities are as intrinsic to the nature of language as expressions in the vocal modality are (e.g., Goldin-Meadow & Brentari, 2015; Özyürek, & Woll, 2019; Perniss et al., 2015). It is possible that the multimodal nature of language and thus its flexible uses through the visual and/or vocal modalities enabled human language faculty to spread around the world so successfully and to adapt to many biologically and socially driven individual, environmental and cultural differences and circumstances. Thus, understanding how expressions not only in vocal but also in the visual modality contribute to our notions of language structure, use, processing, and its transmission (i.e., acquisition, evolution, emergence) in different languages and cultures should be a fundamental goal of language sciences.

Murgiano et al.’s (2020) paper provides an important step in this direction compiling comprehensive evidence and promoting a “language as situated view” as a way to study language from a multimodal perspective and in face-to-face contextual uses. Furthermore the paper opposes this to a “language as system” view defined at the population level and characterized by the use of “arbitrary symbols governed by rules”. In this commentary I will argue that, even though I agree with the general premises of “language as situated view”, such multimodal contextual uses mentioned in the paper can also be systematic and governed by the specific typological characteristics of different languages as well as by culturally defined and grounded communicative conventions. Thus, a strong dichotomy between “systematic” versus “situated” views of language might not be a good characterization of multimodal language if we consider the crosslinguistic variation (and systematicity) in the non-arbitrary and indexical uses of multimodal language in children or in adults (also see Lupyan and Winter, 2018 for systematicity in iconic spoken words).

As also mentioned in the target paper, traditionally, most models, theories and definitions of language have taken specific characteristics of expressions in speech or written text as primary and have emphasized arbitrary, categorical, linear, combinatorial and unichannel nature of language as the core and unique features of language (e.g., Hockett, 1960). These assumptions have also shaped many of our psycholinguistic, neurolinguistic and computational models and approaches to language. Importantly however, visible iconic and indexical (i.e., pointing), simultaneous and multichannel aspects of language specific to expressions in visual modality have been mostly considered not to be core defining features or even sometimes as “fossils” of language evolution (see Jackendoff, 1999 for such a view for gestures). Even though in the field of sign linguistics many aspects of sign languages, such as their arbitrary, categorical, sequential and hierarchical patternings have been considered on a par with spoken languages (Sandler and Lilo-Martin, 2006), those aspects that do not easily fit these definitions have been debated in terms of their linguistic nature (e.g., Cormier, Fenlon, & Schembri, 2015).

As Murgiano et al. (2021) paper points out there is growing evidence showing that especially visible iconic and indexical aspects of both spoken and sign languages are frequent when we investigate language use in face-to-face context and play a fundamental core in language acquisition and processing. This is attributed to their non-systematic nature giving easy and “visible cues” to their meaning. Based on this evidence authors make the claim that we should make a distinction between “language as a system view “defined by arbitrariness and a rule governed system and “language as a situated system view” characterized by use of non-arbitrary symbols having a function in the acquisition and processing of language and that adapting the latter which embeds the former would provide a more inclusive view of language.

However, many findings in the review are reported from a general and “universal” perspective and do not take the crosslinguistic variations in multimodal uses into account (e.g., Kita and Özyürek, 2003; Özyürek et al, 2005, 2008; Özyürek, 2017, 2018a, b; Azar et al, 2020). Based on the latter evidence I would caution first of all making a strong dichotomy between the two views- even though the authors emphasize the situated view embedding the systematic view, albeit without specifying the relations between them. Secondly, I would argue that defining the iconic and indexical uses as described in the paper as “directly” mapping form to referent using a “general and universal cognitive mapping mechanism” and thus facilitating language use, acquisition and processing need to be nuanced.

Even though iconic gestures can reflect aspects of the referent in a motivated way, this is not the same across languages. Crosslinguistic research has shown that speakers’ iconic gestures can be shaped differently in line with the typological differences in how information is packaged across languages (Kita and Özyürek, 2003). For example, while English speakers mostly gesture about a ball rolling down the stairs in one gesture expressing both manner and path of the event, Turkish and Japanese speakers usually depict manner of rolling in one gesture and path only in another one. These differences reflect how events are typologically expressed in different languages, such as in one clause in English and in two clauses in Japanese and Turkish. Furthermore these differences in iconic gestural depictions are not available to children immediately, and are learned as systematic language-specific patterns over time, pointing out that iconicity is not a completely universally accessible feature outside of the specific language it is embedded or integrated in (Özyürek et al., 2008). It has been also found that Turkish-speaking children use iconic gestures much earlier than reported for English speaking children due to using verbs earlier, in line with Turkish being a verb-framed language (Furman, Küntay, Özyürek, 2014). Also, there is growing evidence that pointing gestures are embedded within language-specific demonstrative systems (see Cooperrider et al. (2021) for how pointing is integrated in different ways within signed and spoken language) and they vary in terms of whether they encode spatial characteristic and/or joint attention between speaker and addressee. For example, Turkish speakers are reported to use pointing to referents in context more with one demonstrative (*su*) used when addresses’ attention is not on the referent, than others (*bu, o*) that encode distance. Children have been also found to learn pointing with one demonstrative (*su*) later than with others (*bu,o*). Thus pointing and demonstratives can be coupled in conventional and systematic ways in different languages with different learning trajectories (Peeters and Özyürek, 2016; Küntay and Özyürek, 2006- also see Azar, Backus, Özyürek, 2019 for language specific points to space accompanying pronouns in reference tracking in Turkish discourse). There is also growing research on general multimodal grammars where both visible indexical and iconic ways of communicating are embedded in grammars of different spoken languages (e.g., Floyd, 2016). Furthermore within sign languages, it has been shown that access to sign iconicity is a subjective, culture- specific process tightly linked to signers’ experience with their own sign language (Occhino, Anible, Wilkinson, & Morford, 2017). Finally, there is also crosslinguistic variation in the way iconic structures are recruited in spatial language in different sign languages (Perniss, Zwitserlood, Özyürek, 2015). Novel research is needed to see whether visible iconicity and indexicality universally facilitate acquisition and processing across languages.

Thus as we start widening our view of language as a multimodal system and think of ways to integrate iconic and indexical uses within core language system, we should consider the crosslinguistic variation in this domain from the beginning. Rather than assuming non-arbitrary uses as less systematic and less rule-governed, we should take a crosslinguistic approach to understand systematicity and variation in human language structures, use, processing and transmission based on integration and flexible use of different multimodal semiotic resources- that possibly makes language the adaptive system it is across the globe.

## References

[1] Azar, Z., Backus, A., & Özyürek, A. (2019). General and language specific factors influence reference tracking in speech and gesture in discourse. Discourse Processes, 56(7), 553–574. DOI: 10.1080/0163853X.2018.1519368

[2] Azar, Z., Backus, A., & Özyürek, A. (2020). Language contact does not drive gesture transfer: Heritage speakers maintain language specific gesture patterns in each language. Bilingualism: Language and Cognition, 23(2), 414–428. DOI: 10.1017/S136672891900018X

[3] Cooperrider, K., Fenlon, J., Keane, J., Brentari, D., & Goldin-Meadow, S. (2021). How pointing is integrated into language: Evidence fro speakers and signers. Fronteris in Communication. DOI: 10.3389/fcomm.2021.567774

[4] Cormier, K., Fenlon, J., & Schembri, A. (2015). Indicating verbs in British Sign Language favour motivated use of space. Open Linguistics, 1, 684–707. DOI: 10.1515/opli-2015-0025

[5] Floyd, S. (2016). Modally hybrid grammar? Celestial pointing for time of day reference in Nheengatú. Language, 92(1), 31–64. DOI: 10.1353/lan.2016.0013

[6] Furman, R., Kuntay, A., & Özyürek, A. (2014). Early language-specificity of children’s event encoding in speech and gesture: Evidence from caused motion in Turkish. Language, Cognition and Neuroscience, 29, 620–634. DOI: 10.1080/01690965.2013.824993

[7] Goldin-Meadow, S., & Brentari, D. (2015). Gesture, sign and language: The coming of age of sign language and gesture studies. Behavioral and Brain Sciences, 5, 1–82. DOI: 10.1017/S0140525X15001247 PMC4821822 26434499

[8] Holler, J., & Levinson, S. C. (2019). Multimodal Language Processing in Human Communication. Trends in Cognitive Sciences, 23(8), 639–652. DOI: 10.1016/j.tics.2019.05.006 31235320

[9] Kita, S., & Özyürek, A. (2003). What does cross-linguistic variation in semantic coordination of speech and gesture reveal? Evidence for an interface representation of spatial thinking and speaking. Journal of Memory and Language, 48(1), 16–32. DOI: 10.1016/S0749-596X(02)00505-3

[10] Küntay, A. C., & Özyürek, A. (2006). Learning to use demonstratives in conversation: What do language specific strategies in Turkish reveal? Journal of Child Language, 33(2), 303–320. DOI: 10.1017/S0305000906007380 16826828

[11] Jackendoff, R. (1999). Possible stages in the evolution of language capacity. Trends in Cognitive Sciences, 3(7), 272–279. DOI: 10.1016/S1364-6613(99)01333-9 10377542

[12] Lupyan, G., & Winter, B. (2018). Language is more abstract than you think, or, why aren’t languages more iconic? Philosophical Transactions of the Royal Society B: Biological Sciences, 373(1752), 20170137. DOI: 10.1098/rstb.2017.0137 PMC6015821 29915005

[13] Murgiano, M., Motamedi, Y., & Vigliocco, G. (2020). Situating language in the real-world: the role of multimodal iconicity and indexicality. Journal of Cognition, 3(1), 38. DOI: 10.5334/joc.113 34514309 PMC8396123

[14] Occhino, C., Anible, B., Wilkinson, E., & Morford, J. P. (2017). Iconicity is in the eye of the beholder. How language experience affects perceived iconicity. Gesture, 16(1), 100–126. DOI: 10.1075/gest.16.1.04occ

[15] Özyürek, A. (2017). Function and processing of gesture in the context of language. In R. B. Church, M. W. Alibali, & S. D. Kelly (Eds.), Why gesture? How the hands function in speaking, thinking and communicating (pp. 39–58). Amsterdam: John Benjamins Publishing. DOI: 10.1075/gs.7.03ozy

[16] Özyürek, A. (2018a). Cross-linguistic variation in children’s multimodal utterances. In M. Hickmann, E. Veneziano, & H. Jisa (Eds.), Sources of variation in first language acquisition: Languages, contexts, and learners (pp. 123–138). Amsterdam: Benjamins.

[17] Özyürek, A. (2018b). Role of gesture in language processing: Toward a unified account for production and comprehension. In S.-A. Rueschemeyer, & M. G. Gaskell (Eds.), Oxford Handbook of Psycholinguistics (2nd ed., pp. 592–607). Oxford: Oxford University Press. DOI: 10.1093/oxfordhb/9780198786825.013.25

[18] Özyürek, A., Kita, S., Allen, S., Brown, A., Furman, R., & Ishizuka, T. (2008). Development of cross-linguistic variation in speech and gesture: motion events in English and Turkish. Developmental Psychology, 44(4), 1040–1054. DOI: 10.1037/0012-1649.44.4.1040 18605833

[19] Özyürek, A., & Woll, B. (2019). Language in the visual modality: Cospeech gesture and sign language. In P. Hagoort (Ed.), Human language: From genes and brain to behavior (pp. 67–83). Cambridge, MA: MIT Press.

[20] Peeters, D., & Özyürek, A. (2016). This and that revisited: A social and multimodal approach to spatial demonstratives. Frontiers in Psychology, 7, 222. DOI: 10.3389/fpsyg.2016.00222 26909066 PMC4754391

[21] Perniss, P. M., Özyürek, A., & Morgan, G. (2015). The influence of the visual modality on language structure and conventionalization: Insights from sign language and gesture. Topics in Cognitive Science, 7(1), 2–11. DOI: 10.1111/tops.12127 25565249

[22] Perniss, P. M., Zwitserlood, I., & Özyürek, A. (2015). Does space structure spatial language? A comparison of spatial expression across sign languages. Language, 91(3), 611–641. DOI: 10.1353/lan.2015.0041

[23] Sandler, W., & Lilo-Martin, D. (2006). Sign language and linguistic universals. Cambridge University Press. DOI: 10.1017/CBO9781139163910

